# The Odor Release Regularity of Livestock and Poultry Manure and the Screening of Deodorizing Strains

**DOI:** 10.3390/microorganisms9122488

**Published:** 2021-11-30

**Authors:** Haixia Ma, Feier Li, Evode Niyitanga, Xicun Chai, Shipeng Wang, Yutao Liu

**Affiliations:** 1 College of Artificial Intelligence, Nanjing Agricultural University, Nanjing 210031, China; mahaixia@stu.njau.edu.cn (H.M.); 2020812063@stu.njau.edu.cn (S.W.); 2 Faculty of Mechanical Engineering, Dalian University of Technology, Dalian 116024, China; lifeier@mail.dlut.edu.cn; 3 Faculty of Engineering, Nanjing Agricultural University, Nanjing 210031, China; nvode16@gmail.com (E.N.); 2020212003@stu.njau.edu.cn (X.C.)

**Keywords:** livestock and poultry manure, source suppression, end treatment, deodorization strains

## Abstract

Human living environments and health are seriously affected by the odor produced from fermentation of livestock and poultry manure. In order to reduce the odor pollution caused by livestock and poultry manure, efficient strains were screened and two methods were tried in this study. The orthogonal test design was used to analyze the gas produced by pig manure under different conditions of temperature, time, wheat straw doping amount and calcium carbonate doping amount. Then, according to ammonia, hydrogen sulfide and comprehensive odor removal effects, the high efficiency of deodorizing strains were screened. The results showed that pig manure produced the least odor when the temperature was 20 °C, added 0% calcium carbonate, 20% wheat straw and waited for 48 h. Three strains were screened to inhibit the odor production of pig manure: *Paracoccus denitrificans*, *Bacillus licheniformis* and *Saccharomyces cerevisiae*, showed that their highest removal rate of ammonia and hydrogen sulfide gas could reach 96.58% and 99.74% among them; while for three strains of end-control pig manure stench: *Pichia kudriavzevii*, *P. denitrificans* and *Bacillus subtilis*, the highest removal rate of ammonia and hydrogen sulfide gas reached 85.91% and 90.80% among them. This research provides bacteria resources as the high-efficiency deodorizing function for the source suppression and the end treatment of the odor gas of pig manure, which has high application value for the control of odor pollution.

## 1. Introduction

With the increasing consumption of meat, eggs, and milk, the poultry and livestock breeding industry has developed rapidly [1]. This was followed by large livestock manure production, generating a large amount of malodorous gas due to the accumulation of livestock and poultry manure; this accumulation cannot be processed in time, which seriously pollutes the environment [2]. Prolonged exposure to such gas can cause different health problems like headache, nausea, convulsions and other adverse health reactions, which have a serious impact on the lives of residents [3,4]. Therefore, it is urgent to solve the problem of odor pollution caused by livestock and poultry manure.

The production of malodorous gas is the result of the decomposition of carbohydrates, protein and other components in livestock and poultry manure by microorganisms [5,6]. Many scholars have done research on the distribution and diffusion of malodorous gas. For example, Maïzi et al. used computational fluid dynamics (CFD) models to predict the concentrations of odorous gases [7]; Kim et al. [8] found that the diffusion of odorous gases in pig farms was related to factors such as wind speed and atmospheric stability. The spatial influence of odor has been well explained by previous studies, however, there are only a few research investigations into the production and release of odor in livestock and poultry manure under the combined conditions of different external factors. Therefore, it is necessary to carry out work in this area, in order to provide a basis for the selection of suitable microbial agents and the time of use.

Management of malodorous gas in livestock and poultry manure is based on different technological intensive methods. The removal methods are divided into three main methods such as: physical, chemical and biological methods [9]. The bioscrubber and biofiltration methods and so on are the most widely used technologies of microbial deodorization [10]. However, the removal efficiency of the bioscrubber method is affected by water-soluble solvents and the operation cost is high, while the reaction conditions of biofiltration are difficult to control [11,12]. Compared with the bioscrubber method, the biofiltration method and other odor ectopia treatment technologies, the use of microbial agents for source treatment has the advantages of low cost, simple operation, and no need for special equipment; microbial agents have the function of efficient adsorption and transformation of odorous substances [13,14]. It was found that methionine (C_5_H_11_O_2_NS) added to poultry feed with a disease prevention effect could lead to a large amount of N and S excreted by poultry, so ammonia and sulfur-containing compounds were the main components of malodorous gas from livestock and poultry manure [15,16,17]. Three optimization strains with relatively higher deodorizing capacity were isolated from landfill leachate by Zeng et al. which were identified as *Bacillus megaterium*, *Lactobacillus acidophilus* and *Alcaligenes faecalis* respectively; when the mixed inoculation rate was 1:1.5:0.5, the removal rates of ammonia and hydrogen sulfide were 83.56% and 70.25%, respectively [18]. The strain of *Lactobacillus plantarum* was screened and isolated by Kim et al. from piggery mud samples, and the removal efficiency of ammonia reached a maximum of 98.5% after 50 h of incubation [19]. The compound microbial deodorant prepared by *Bacillus megaterium*, *Candida tripicalis* and *Streptomyces griseus* could remove more than 80% of the odor from chicken, pig and cow manure, and more than 65% of the hydrogen sulfide could be removed [20]. This showed that microorganisms could effectively remove the malodourous gas produced by livestock and poultry manure. However, the study above just focused on the management of malodorous after its production, and the microbial agents at present need a very long time to deal with the gas, so it is necessary to screen microorganisms that can effectively inhibit the production of malodorous gas at the source and efficiently adsorb and transform malodorous gas at the end quickly.

In order to grasp the release rules of malodorous gas in livestock and poultry manure, screen the microbial strains that inhibit the production of malodorous gas in livestock and poultry manure and efficiently transform the malodorous gas, pig manure was used as research material, while ammonia, hydrogen sulfide and comprehensive malodorous gases were used as indicators to study the production rules of malodorous gas from pig manure under different external conditions. The microbial strains with effective inhibition at the source and efficient adsorption transformation at the end were screened, which provided material and technical support for the healthy development of the livestock and poultry breeding industry and odor control.

## 2. Materials and Methods

### 2.1. Experiment Material

Pig manure and wheat straw were provided by Modern Farm in Rugao, Jiangsu Province; Bacteria B1, B2.2.2, B2.4, B2.6, B3.1 and *B. licheniformis* (It had been identified and registered by Guangdong Microbial Species Preservation Center with the serial number GDMCC No: 61879) were isolated from the sandy soil of livestock and poultry farms; other strains were provided by the laboratory for cultivation. The physicochemical properties of pig manure and wheat straw used in the experiment are shown in Table 1.

### 2.2. The Culture of Deodorizing Microorganisms

The PDA (Potato Dextrose Agar) media was prepared as follows: removed the peel of 200 g potatoes and cut them into small pieces of 1 cm^3^ in size, then boiled them in deionized water for 30 min, filtered them with 8 layers of gauze and added 20 g glucose and kept the volume to 1 L. The LB (Luria-Bertani) media was prepared by using 10.0 g/L tryptone, 5.0 g/L yeast extract, 5.0 g/L NaCl and 1 mL 1 N NaOH, and the pH was set to 7.0. The *P. denitrificans* culture medium was prepared in the ratio of 5.0 g/L (NH_4_)_2_SO_4_, 0.5 g/L MgSO_4_·7H_2_O, 0.7 g/L KH_2_PO_4_, 0.5 g/L CaCl_2_·2H_2_O, and the pH range was set to 6 to 7. The *Actinomycetes* media was prepared by using 10.0 g/L soluble starch, 1.0 g/L peptone, 1.0 g/L KNO_3_, 0.2 g/L K_2_HPO_4_, 0.3 g/L MgSO_4_, 0.5 g/L NaCl, 1.0 g/L CaCO_3_, 0.5 g/L yeast, 2.0% (*w*/*v*) agar and the pH was set to 7.8. The MRS (Man Rogosa Sharpe) media was prepared as follows: weighed 54 g of MRS broth and added 1 L of deionized water, stirred, heated and boiled until completely dissolved. All prepared media were autoclaved at 121 °C for 20 min.

An amount of 0.1 mL liquid of *P. kudriavzevii* (it had been identified and registered by the Guangdong Microbial Species Preservation Center with the serial number GDMCC No: 61876) and *S. cerevisiae* (it had been identified and registered by the Guangdong Microbial Species Preservation Center with the serial number GDMCC No: 61877) were inoculated into PDA media, mixed evenly, and shaken at 30 °C for 72 h; in the same way, Bacteria 2, *B. subtilis* (it had been identified and registered by the Guangdong Microbial Species Preservation Center with the serial number GDMCC No: 61878), B1, *B. licheniformis*, B2.2.2, B2.4, B2.6 and B3.1 were added to the LB media, and *P. denitrificans* (it had been identified and registered by Guangdong Microbial Species Preservation Center with the serial number GDMCC No: 61875) and *Actinomycetes* were added to corresponding media, respectively, and shaken at 30 °C for 72 h; 0.1 mL *Plant lactobacillus* was inoculated into MRS media and placed in a light incubator (GZP-250N, Shanghai Senxin Experimental Instrument Co., Ltd., Shanghai, China) at 28 °C for 24 h.

### 2.3. Experiment on the Odor Production of Pig Manure

#### 2.3.1. Single Factor Experiment

The purpose of this part is to explore the variation of malodorous gas of livestock and poultry manure with a single factor; pig manure was selected as the substrate, the ratio of ingredients was adjusted according to the physical and chemical properties, calcium carbonate and wheat straw were added, and then the mixture was evenly mixed and sealed with a rubber plug. This mixture was then put into a constant temperature shaker (TS-2403CL, Wuxi Maret Technology Co., Ltd., Wuxi, China) and the odor value was measured every 24 h with a portable stench analyzer (PAir2000-EFF-B, Beijing Big Dipper Institute of Industrial Chemistry, Beijing, China).

Single factor experiments lasting for 240 h were carried out at different temperatures (20 °C, 35 °C and 45 °C, the doping amount of calcium carbonate was 0.5% of dry matter content of pig manure, and the doping amount of wheat straw was 15% of dry matter content of pig manure). Different doping amounts of calcium carbonate (0%, 0.5% and 1%, the temperature was 35 °C, and the doping amount of wheat straw was 15% of the dry matter content of pig manure). Different doping amounts of wheat straw were used (0%, 15% and 20%, the temperature was 35 °C, and the doping amount of calcium carbonate was 0.5% of dry matter content of pig manure), to investigate the production rule of malodorous gas with three single factors and the variation of malodorous gas of pig manure with time under the conditions of 35 °C. The doping amount of calcium carbonate was 0.5% of dry matter content of pig manure and the doping amount of wheat straw was 15% of dry matter content of pig manure.

#### 2.3.2. Orthogonal Experiment

In order to further find the conditions to minimize the intensity of odor release, temperature, the calcium carbonate doping amount, the wheat straw doping amount and time were selected as factors according to the results of the single factor experiment, and combined with the actual situation. Three different levels of these four factors were selected respectively. The design factor level table is shown in Table 2.

### 2.4. Screening of High-Efficiency Anti-Odor Microbial Strains for Spraying

The same quality of pig manure was placed in plastic bottles, and after a thorough mixing of the pig manure; 50 mL of each bacterial solution was centrifuged (the time and revolution were set at 10 min and 10,000 r/min, respectively), and 100 mL of deionized water was mixed evenly with the centrifuged bacterial solution. Sterile water was used as a blank control, and both the deionized water and the bacterial solution were quantitatively sprayed on the surface of pig manure; sealed with a rubber plug after spraying; the pig manure was placed in a constant temperature shaker for 5 h; a stench analyzer was then used to measure the concentration of odors inside the plastic bottles. The comprehensive odor concentration is dimensionless, and its data processing formula is as follows:

W = H/60,(1)

in the formula, W is the comprehensive odor concentration after treatment, and its unit is per gram of dry manure. H is the measured comprehensive odor concentration value, dimensionless.

### 2.5. Screening of Microbial Strains for Terminal Absorption of Malodorous Gases

A 2 L air collecting bag was connected to the outlet of portable stench analyzer to collect odor and record the value of each odor index in the collection process; next, the bacteria liquid was diluted with sterile water in a ratio of 1:2 and it was poured into an empty plastic bottle. Then the odor in the air collecting bag was injected into the bacterial liquid; the change of the odor index was recorded and the deodorization rate of the bacteria solution to each index was calculated to screen out effective bacteria. The formula for the deodorization rate is as follows:

S = (T − t)/T × 100,(2)

where S is deodorization rate, its unit is %; T is the odor concentration value before entering the bacterial solution, and its unit is ppm; and T is the odor concentration value (ppm) of the bacteria solution.

### 2.6. Data Processing

The trials in this study had three replicates. Data processing was performed using Excel 2016 (Microsoft Corporation), SPSS Statistics 25.0 (IBM Corporation, New York, NY, USA) and so on. Significance was assessed using Duncan’s test (α = 0.05) for multiple comparisons.

## 3. Results

### 3.1. Single Factor Experiment on Odor Producing Rule of Pig Manure

**Figure 1:**
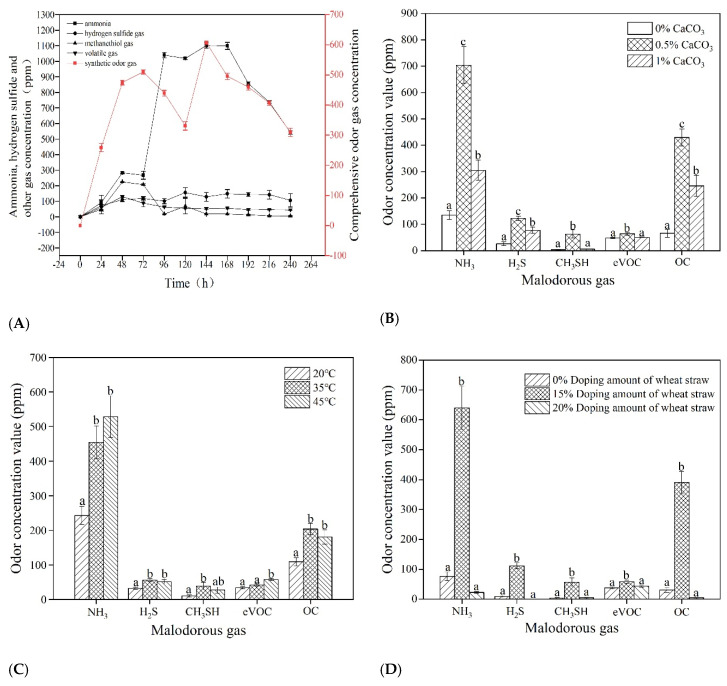
Production regularity of malodorous gas of pig manure with different factors: (**A**) The production regularity of malodorous gas in pig manure at different times; (**B**) The rule of odor gas generation in pig manure with different calcium carbonate doping amounts; (**C**) Production rules of malodorous gases of pig manure at different temperatures; and (**D**) The rule of odor gas generation of pig manure with different wheat straw doping amounts. Different lowercase letters above the bars indicate significant differences (*p* < 0.05).

Temperature, wheat straw, time and calcium carbonate were selected as experimental factors to observe the changes in the concentration of five malodorous gases for better understanding of the changes in the level of malodorous gases produced by pig manure. The comprehensive odor concentration increased rapidly at the early stage of pig manure fermentation, and reached 474.51 per gram of dry manure within 48 h; at 72 h, it reached the peak of 510.23 per gram of dry manure. The comprehensive odor concentration decreased to 309.75 per gram of dry manure at 240 h, but still maintained a high level, and had the characteristics of continuous release. Ammonia had the characteristics of less release in the early stage and large release in the mid-term, which was gradually decreased in the later period, and it continued to be near the peak value from 96 to 168 h. Hydrogen sulfide, methanethiol and volatile gases had the characteristics of low release in the early stage and gradually declined after reaching the peak value with weak persistence (Figure 1A). The five malodorous gas indexes all reached the maximum value when the doping amount of calcium carbonate was 0.5% and the minimum value when the doping amount of calcium carbonate was 0%, which indicated that a certain amount of calcium carbonate could promote the odor production of pig manure (Figure 1B). We had thought the addition of calcium carbonate would neutralize pH to alleviate the acidification which might occur in livestock and poultry manure during the composting process, and then inhibited odor emission, yet the result did not confirm our expectations; why the 0.5% doping amount had significant promotion effect needed to be further clarified. Methanethiol, hydrogen sulfide and comprehensive malodorous gases released the highest concentration at 35 °C, ammonia and volatile gases released the highest intensity at 45 °C, and all released less gas at 20 °C, indicating that appropriate temperature control can inhibit the generation of malodorous gas (Figure 1C). This was consistent with the conclusion of Le et al. [21], that temperature increase would lead to an increase in odor emissions, and reducing the temperature and ventilation rate would reduce the emission of odor gases from pig manure. When the wheat straw doping amount was 15%, all the five malodorous gas indexes reached the maximum value, and less malodorous gas was released when the doping amount of wheat straw was 0% or 20%, indicating that the addition of a certain amount of wheat straw could promote the odorous production of pig manure (Figure 1D).

### 3.2. Orthogonal Experiment on Odor Producing Rule of Pig Manure

The purpose of this part of the study was to obtain the optimal conditions for the least odor production of pig manure. Therefore, the orthogonal experiment was carried out on the basis of a single factor experiment, time was taken as a factor and three levels were selected according to the results of single factor experiment. The results of the orthogonal experiment are shown in Table 3, and the results of the range analysis are shown in Table 4. The data in the table are the odor production values of each factor at each level. The greater the range means the greater the influence of this factor on the test index. The data in the table show that the influence of each factor on the ammonia production of pig manure from large to small are D, C, A, B; the influence of the factors on hydrogen sulfide are C, D, B, A from large to small; the influence on methanethiol on the factors are D, A, C, B in descending order; the effects on volatile organic compounds from large to small are D, C, A, B; and the influence of comprehensive odor concentration are C, D, B, A from large to small. The results indicate that the time and the amount of wheat straw have a great influence on the stench of pig manure, and the stench could be reduced by controlling the time of piling pig manure or adding a certain amount of wheat straw. According to the range analysis results in Table 4, the combinations with the lowest concentrations of ammonia, hydrogen sulfide, methanethiol, volatile organic compounds and comprehensive odor are A_1_B_1_C_2_D_1_, A_1_B_1_C_3_D_1_, A_2_B_2_C_3_D_3_, A_2_B_2_C_3_D_2_ and A_1_B_1_C_3_D_1_, respectively, based on the test results of ammonia, hydrogen sulfide and comprehensive malodorous gases. The combination with the least odor production was determined as A_1_B_1_C_3_D_1_, that is, under the condition of adding 0% calcium carbonate and 20% wheat straw at the temperature of 20 °C, the odor production is the least on the second day and long-term stacking of pig manure should be avoided as far as possible.

### 3.3. Different Deodorant Effects of Microbial Spray

Spray deodorization can not only effectively adsorb odor molecules in the air, but also inhibit the odor production of pig manure from the source. This method uses a small amount of deodorizing treatment, at low cost, and in the case of appropriate selection of bacteria liquid can effectively deodorize.

**Figure 2:**
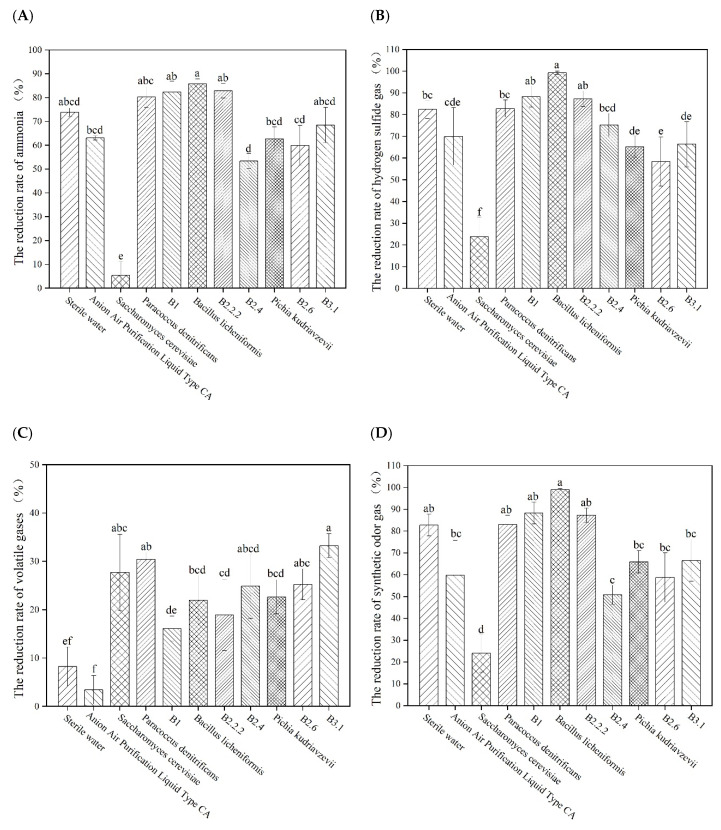
The removal rate of malodorous gas by different bacterial liquids: (**A**) The removal rate of ammonia gas by different bacteria; (**B**) The removal rate of hydrogen sulfide gas by different bacterial liquids; (**C**) The removal rate of volatile gases by different bacterial liquids; and (**D**) The removal rate of different bacterial liquids on the integrated malodor concentration. Different lowercase letters above the bars indicate significant differences (*p* < 0.05).

In this experiment, 10 kinds of microorganisms were selected for screening, and sterile water was used as a blank control. The results showed (Figure 2A,B) that *P. denitrificans*, B1, *B. licheniformis*, and B2.2.2 were not significantly different from the control treatment (*p* > 0.05). However, compared with the control treatment, the removal rates of *P. denitrificans*, B1, *B. licheniformis* and B2.2.2 were all above 80%, and the removal rates were 80.25%, 82.30%, 85.81%, 82.91%, respectively, while the removal rates of other strains were all below 80%. It was seen that *B. licheniformis* had the strongest ability to remove ammonia. B1, *B. licheniformis* and B2.2.2 had a strong ability to remove hydrogen sulfide gas in comparison with the control treatment, and there were significant differences with the control treatment (*p* < 0.05). The highest removal rate of *B. licheniformis* was 99.29%, which greatly reduced the concentration of hydrogen sulfide gas, and was about 8% higher than the deodorization device designed by Huang et al. [22]. The removal rate of hydrogen sulfide gas by *P. denitrificans* was also above 80%, but there was no significant difference with that of the control treatment (*p* > 0.05). It can be seen from Figure 2C that, except for Anion air purification liquid, the volatile gas removal rates of other strains are higher than that of the control treatment, and the removal rates of B2.4, B2.6, B3.1, *S. cerevisiae* and *P. denitrificans* are significantly different from those of other strains (*p* < 0.05). *S. cerevisiae* had no obvious effect in removing other gases, but it had an outstanding effect in removing volatile gases, with a removal rate of 27.70%. The removal effect of *P. denitrificans* on volatile gas was also very good, and the removal rate reached 30.43%. Compared with the control treatment, *P. denitrificans*, *B. licheniformis*, B2.2.2 and B1 had a stronger ability to remove comprehensive odor, and the removal rates were 82.97%, 98.93%, 87.26% and 88.31%, respectively; *B. licheniformis* had the best removal effect among them. The removal effect of other strains was not as good as that of the control treatment. Therefore, four strains with high efficiency in removing comprehensive odor gas were obtained (Figure 2D).

Based on the above analysis, *B. licheniformis* has a good removal effect on the four odor indexes. *P. denitrificans* also has a high ability in removing the concentration of the four gases. *S. cerevisiae* is very effective in removing volatile gases. Therefore, three strains were selected for spray deodorization.

### 3.4. Deodorization Effect of Different Microbial End Treatments

**Figure 3:**
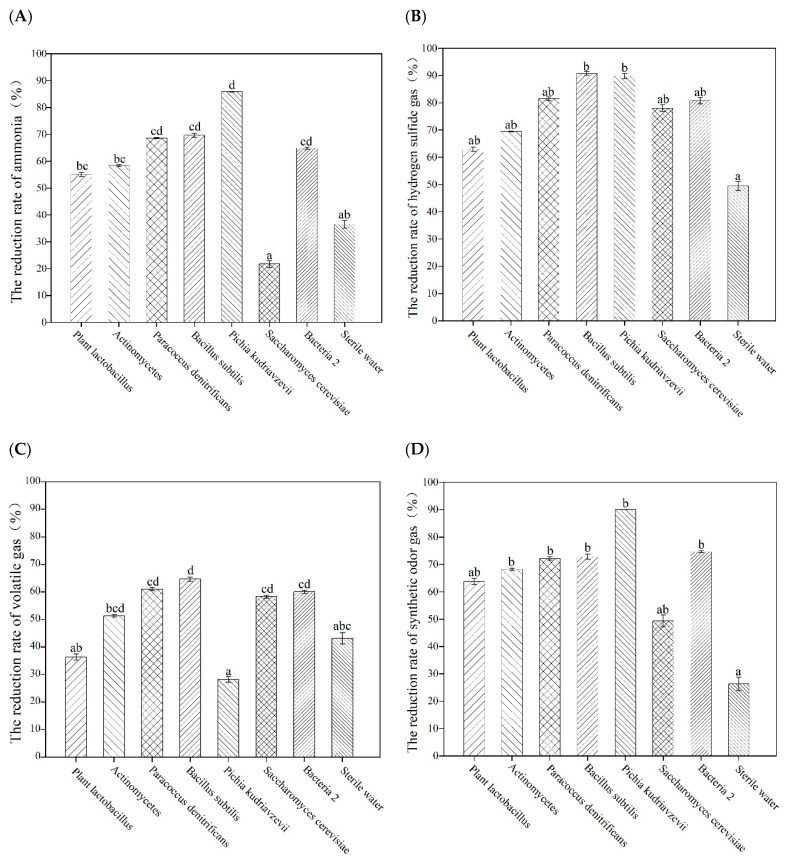
The removal rate of malodorous gas by different bacterial liquids: (**A**) The removal rate of ammonia gas by different bacteria; (**B**) The removal rate of hydrogen sulfide gas by different bacterial liquids; (**C**) The removal rate of volatile gases by different bacterial liquids; (**D**) The removal rate of different bacterial liquids on the integrated malodor concentration. Different lowercase letters above the bars indicate significant differences (*p* < 0.05).

The removal rate of deodorization technology used for source control will not be 100%, so further removal by end treatments is required. The removal of ammonia, hydrogen sulfide, volatile and other gas concentrations by 7 kinds of microorganisms were studied in this experiment. The highest removal rates of ammonia and hydrogen sulfide gas by microorganisms were 85.91% and 90.80% respectively (Figure 3A,B); all strains except *S. cerevisiae* had a stronger ability to remove ammonia compared with blank control sterile water, among which *P. kudriavzevii* had the highest ammonia removal rate of 85.91%, while *P. denitrificans*, *B. subtilis* and Bacteria 2 had no significant difference with *Pichia kudriavzevii* (*p* > 0.05), and the removal rates of ammonia were 68.03%, 69.69% and 64.90%, respectively. However, compared with *P. kudriavzevii*, *P. lactobacillus* and *Actinomycetes* had significant differences (*p* < 0.05). According to the analysis, four strains with high efficiency in ammonia removal were obtained. Compared with the control treatment, the selected microbial strains had higher abilities to remove hydrogen sulfide gas, and there was no significant difference among all treatments (*p* > 0.05), but *B. subtilis* had the strongest ability to remove hydrogen sulfide gas, reaching 90.80%. The removal rates of hydrogen sulfide by *P. kudriavzevii*, *P. denitrificans* and Bacteria 2 were 89.79%, 81.48% and 80.77%, respectively. This was about 20% higher than the degradation rate of hydrogen sulfide by *Bacillus flexus*, a deodorizing microorganism selected from pig manure by Shen Qi et al. [23]. Therefore, four strains that efficiently removed hydrogen sulfide gas could be obtained.

According to Figure 3C, all strains except *P. lactobacillus* and *P. kudriavzevii* had a strong removal effect on volatile gas, and there was significant difference with the control treatment (*p* < 0.05). The removal rate of volatile gas by *B. subtilis* was the highest, reaching 64.74%. The volatile gas removal rates of Bacteria 2 and *P. denitrificans* were also above 60% (60.10% and 61.04%, respectively), and three strains with high volatile gas removal efficiency were obtained. As can be seen from Figure 3D, compared with the control treatment, the selected strains have a stronger ability to remove comprehensive odor concentration, and there is no significant difference among strains (*p* > 0.05). *P. kudriavzevii* had the highest removal rate of comprehensive odor gas (90.01%). The removal rates of *B. subtilis*, *P. denitrificans*, Bacteria 2 and *Actinomycetes* were 72.91%, 72.15%, 74.66% and 68.24%, respectively. There was no significant difference in the removal rate of *P. lactobacillus* and *S. cerevisiae* from the blank control (*p* < 0.05). Five strains with a high removal rate of comprehensive odor concentration were obtained.

Since ammonia and hydrogen sulfide gases are the main odor components, priority should be given to the removal rate of these two gases while selecting strains. Based on the above analysis, *B. subtilis*, *P. denitrificans* and *P. kudriavzevii* are selected as the end-absorbing odor gas strains.

## 4. Discussion

### 4.1. Factors Affecting the Release of Malodorous Gases from Pig Manure

Controlling the stacking conditions of livestock and poultry manure can reduce the production of malodorous gas. Previous studies have found that composting temperature, ventilation mode, initial moisture content and raw material mixing ratio significantly affect aerobic composting [24,25]. The pig slurry with straw husks was proven to reduce hydrogen sulfide gas emissions [26]; Ding Gangqiang et al. [27] found that the nitrogen-containing gas in the solid stacking process of pig manure was mainly discharged in the form of ammonia, and the nitrogen-containing gas emission of the pile covered with rice straw was lower than that of uncovered; which proved that the addition of straw could promote the composting effect and reduce the emission of pig manure odor, but the effect of adding different proportions was not seen. However, we found that the pig manure odor production with 15% wheat straw was higher than that without wheat straw, and the production of ammonia, hydrogen sulfide and comprehensive odor gas when the addition ratio was 20% was lower than 0%, which was consistent with the conclusions of some studies by Tian Chunyan et al. [28], indicating that the ratio of straw to feces has a more important impact on odor production. Therefore, in the comprehensive consideration of composting effect and odor-producing intensity control, it is necessary to choose the appropriate straw addition proportion.

Temperature and time are also important factors in odor emissions. Zhu Wei et al. [29] found in their study on the aerobic fermentation process of sheep feces that environmental temperature had a very significant impact on the production of harmful gases in feces fermentation. When the temperature was 30–35 °C, the emissions of ammonia and hydrogen sulfide were higher than emissions produced when the temperature was 10–15 °C and 20–25 °C, which was consistent with the conclusion that the emission of malodorous gases from pig feces was higher than that of 20 °C, at 35 °C in this study. However, it was further found that too high a temperature would reduce the odor production. The methanethiol and comprehensive odor gas production at 45 °C was significantly lower than that at 35 °C, which might be caused by the decreased activity of fecal odor-producing microorganisms caused by the excessive temperature. It was also found in this study that this was similar to the odor release characteristics of pig manure at different fermentation stages discovered by predecessors [30]. Therefore, only by mastering the change of odor during aerobic fermentation can we take measures to reduce the air pollution caused by aerobic fermentation.

### 4.2. Source Deodorization and End Treatment of Microbial Strains

Long-term exposure to malodorous gases has adverse effects on health [31]. Compared with other technologies, microorganisms have the advantages of decomposition of odor molecules, less secondary pollution and better persistence [32]. The key of the microbial deodorization method is to select efficient deodorization microorganisms. The *Lactobacillus strain* isolated by Yan et al. [33] could reduce ammonia and hydrogen sulfide gas by more than 30%; Sun Xiaojun et al. [34] obtained that the degradation rate of ammonia nitrogen of 53.19% by the compound agent composed of *Bacillus flexus* and *Lactobacillus*. At present, there are few microorganisms with efficient deodorization effect, and most of them can only remove a single gas. In this study, we screened out the microbial strains that were suitable for spraying and other sources to inhibit odor production and the microbial strains that adsorbed and transformed odor at the end. The highest reduction rates of *P. denitrificans*, *B. subtilis*, *P. kudriavzevii* and *B. licheniformis* to ammonia and hydrogen sulfide gas were 80.25%, 69.69%, 85.91%, 85.81% and 82.80%, 90.80%, 89.79%, 99.29%, respectively. Compared with the strains obtained in the above study, the odor removal effect was better. Moreover, studies had found that the reduction rate of ammonia and hydrogen sulfide gas after terminal absorption was about 10% lower than that after spraying, which might be related to the solubility of gas in the absorption solution [35]. The multi-step handling method should be effective to manage odor at very low-level. In the initial stage of livestock manure accumulation, microorganisms used for spraying play a role in inhibiting the production of malodorous gas; when excessive odor is still generated after spraying, the odor will be further removed by means of end absorption. The end absorption takes place in a large space and will not reach the saturation state of microorganisms at a time, so the microorganisms have a chance to renew and the odor can be continuously removed. However, the specific odor value that makes the microorganisms saturated at one time still needs to be further studied.

The odor components are complex, and a single strain often cannot achieve the removal of multiple odor components. The *B. licheniformis* obtained in this study has a high degradation rate of ammonia and hydrogen sulfide, but the degradation rate of volatile organic compounds is not as good as other strains. On the contrary, *S. cerevisiae* has the function of removing volatile gas efficiently. Therefore, in the future, the ability of different microbial strains to remove different odor components should be fully considered, and the effects of different microbial combinations and proportions on odor removal should be studied, so as to develop efficient deodorant agents and apply them to spray or adsorption deodorant devices. In addition to improving the efficiency of a single deodorization method, a combination of several methods should be considered to achieve higher efficiency.

## 5. Conclusions

(1) The release intensity of malodorous gas from livestock and poultry feces is related to temperature, doping amount of calcium carbonate, doping amount of wheat straw and time. Among them, the wheat straw doping amount and time had the greatest influence: when the wheat straw doping amount was 15%, the concentration of five odorous gases reached the maximum value; the comprehensive odor gas concentration in the middle of pig manure fermentation was large and persistent; ammonia gas had the characteristics of low release in the early stage, large release in the middle stage and gradually decreased in the late stage; hydrogen sulfide, methanethiol and volatile gas had the characteristics of low release in the early stage, gradually declined after reaching the peak, and not a strong persistence. The doping amount of calcium carbonate and temperature have little effect on the release of odorous gas.
(2) *B. licheniformis*, *P. denitrificans* and *S. cerevisiae* had outstanding effects on inhibiting odor production at source. The reduction rates of *B. licheniformis* on ammonia and hydrogen sulfide were 85.81% and 99.29%, respectively; the reduction rates of ammonia and hydrogen sulfide by *P. denitrificans* were 80.25% and 82.80%, respectively. Compared with other strains, *S. cerevisiae* had a significant effect on volatile gas removal, with a reduction rate of 27.7%.
(3) *P. kudriavzevii*, *P. denitrificans* and *B. subtilis* can be used as effective end-absorbing and transforming odorous gas strains. The reduction rates of ammonia and hydrogen sulfide by *P. kudriavzevii* were 85.91% and 89.79%, respectively; *B. subtilis* had a strong ability to degrade hydrogen sulfide gas, and the reduction rates of ammonia gas and hydrogen sulfide gas were 69.69% and 90.80%, respectively; the reduction rates of ammonia and hydrogen sulfide by *P. denitrificans* were 68.03% and 81.48%, respectively.

## Figures and Tables

**Table 1:** Physicochemical properties of materials.

The Name of Material	TS (%)	VS (%)	ω (%)	C (%)	N (%)
pig manure	38.20	91.79	61.80	29.02	3.20
wheat straw	93.20	91.70	6.80	40.26	1.17

Note: C and N contents are the percentage of dry matter in raw materials.

**Table 2:** Factors and levels of orthogonal experiment.

Level	A/°C	B/%	C/%	D/h
1	20	0	0	48
2	35	0.5	15	96
3	45	1	20	144

Note: A, B, C and D respectively represent temperature, calcium carbonate doping amount, wheat straw doping amount and time, and the same below.

**Table 3:** The results of the orthogonal experiment.

Test No.	A	B	C	D	Empty Row	NH_3_/ ppm	H_2_S/ ppm	CH_3_SH/ppm	eVOC/ppm	OC
1	1	1	1	1	1	68.94	19.60	33.70	89.90	5477.00
2	1	1	1	1	2	242.80	139.70	118.00	129.50	32,452.00
3	1	1	1	1	3	16.41	0.00	70.80	96.00	3431.00
4	1	2	2	2	1	418.40	99.50	4.95	41.13	17,946.00
5	1	2	2	2	2	396.70	103.70	4.42	43.26	17,995.00
6	1	2	2	2	3	431.30	77.09	3.37	40.82	14,564.00
7	1	3	3	3	1	16.62	0.00	0.00	41.52	24.30
8	1	3	3	3	2	140.00	22.40	1.74	45.65	4314.00
9	1	3	3	3	3	83.41	11.72	0.00	44.13	2959.00
10	2	1	2	3	1	103.20	3.94	1.09	50.18	893.60
11	2	1	2	3	2	187.90	14.69	2.89	44.61	2830.00
12	2	1	2	3	3	116.00	10.12	1.10	51.79	2045.00
13	2	2	3	1	1	38.99	0.00	0.47	45.17	88.01
14	2	2	3	1	2	35.94	0.63	0.44	44.78	80.69
15	2	2	3	1	3	34.93	0.00	0.28	44.04	67.84
16	2	3	1	2	1	991.10	177.70	0.00	51.44	41,352.00
17	2	3	1	2	2	1072.00	192.60	0.00	61.23	30,571.00
18	2	3	1	2	3	801.30	191.60	1.96	64.58	41,632.00
19	3	1	3	2	1	874.50	4.97	18.30	64.06	3139.00
20	3	1	3	2	2	112.20	0.00	18.40	60.53	995.30
21	3	1	3	2	3	1141.00	183.80	18.50	76.90	28,259.00
22	3	2	1	3	1	1135.00	192.10	18.20	74.07	38,635.00
23	3	2	1	3	2	1140.00	183.30	18.30	77.46	39,727.00
24	3	2	1	3	3	1132.00	166.20	18.60	78.59	31,355.00
25	3	3	2	1	1	13.46	0.00	84.60	104.80	4120.00
26	3	3	2	1	2	12.94	0.00	96.10	112.20	4690.00
27	3	3	2	1	3	9.98	0.00	68.70	103.90	3317.00

**Table 4:** Results of the significance test for range analysis.

Evaluation Index		A	B	C	D
NH_3_/ppm	$\bar{K}$ _1_	201.62	318.11	733.28	52.71
	$\bar{K}$ _2_	375.71	529.25	187.76	693.17
	$\bar{K}$ _3_	619.01	348.98	275.29	450.46
	R	417.39	211.15	545.52	640.46
H_2_S/ppm	$\bar{K}$ _1_	52.63	41.87	139.34	17.77
	$\bar{K}$ _2_	63.62	92.50	34.34	112.47
	$\bar{K}$ _3_	82.26	64.15	24.84	68.27
	R	29.63	50.63	114.51	94.70
CH_3_SH/ppm	$\bar{K}$ _1_	26.33	31.42	31.06	52.57
	$\bar{K}$ _2_	0.91	7.67	29.69	7.77
	$\bar{K}$ _3_	39.97	28.12	6.46	6.88
	R	39.05	23.75	24.60	45.69
eVOC/ppm	$\bar{K}$ _1_	63.55	73.72	80.31	85.59
	$\bar{K}$ _2_	50.87	54.37	65.85	55.99
	$\bar{K}$ _3_	83.61	69.94	51.86	56.44
	R	20.07	19.35	28.44	29.59
OC	$\bar{K}$ _1_	11,018.03	8835.77	29,403.56	5969.28
	$\bar{K}$ _2_	13,284.46	17,828.73	7600.07	21,828.14
	$\bar{K}$ _3_	17,137.48	14,775.48	4436.35	13,642.54
	R	6119.44	8992.96	24,967.21	15,858.86

Note: $\bar{K}$_1_–$\bar{K}$_3_ respectively represent the average value of each factor at each level; R stands for range.

## Data Availability

The data that support the findings of this study are available from the corresponding author upon reasonable request.
